# Targeted SNP discovery in Atlantic salmon (*Salmo salar*) genes using a 3'UTR-primed SNP detection approach

**DOI:** 10.1186/1471-2164-11-706

**Published:** 2010-12-15

**Authors:** Rune Andreassen, Sigbjørn Lunner, Bjørn Høyheim

**Affiliations:** 1 Faculty of Health Sciences, Oslo University College, Oslo, Norway; 2 Norwegian School of Veterinary Science, BasAM-Genetics, PO Box 8146 DEP, NO-0033 Oslo, Norway

## Abstract

**Background:**

Single nucleotide polymorphisms (SNPs) represent the most widespread type of DNA variation in vertebrates and may be used as genetic markers for a range of applications. This has led to an increased interest in identification of SNP markers in non-model species and farmed animals. The *in silico *SNP mining method used for discovery of most known SNPs in Atlantic salmon (*Salmo salar*) has applied a global (genome-wide) approach. In this study we present a targeted 3'UTR-primed SNP discovery strategy that utilizes sequence data from *Salmo salar *full length sequenced cDNAs (FLIcs). We compare the efficiency of this new strategy to the *in silico *SNP mining method when using both methods for targeted SNP discovery.

**Results:**

The SNP discovery efficiency of the two methods was tested in a set of FLIc target genes. The 3'UTR-primed SNP discovery method detected novel SNPs in 35% of the target genes while the *in silico *SNP mining method detected novel SNPs in 15% of the target genes. Furthermore, the 3'UTR-primed SNP discovery strategy was the less labor intensive one and revealed a higher success rate than the *in silico *SNP mining method in the initial amplification step. When testing the methods we discovered 112 novel bi-allelic polymorphisms (type I markers) in 88 salmon genes [dbSNP: ss179319972-179320081, ss250608647-250608648], and three of the SNPs discovered were missense substitutions.

**Conclusions:**

Full length insert cDNAs (FLIcs) are important genomic resources that have been developed in many farmed animals. The 3'UTR-primed SNP discovery strategy successfully utilized FLIc data to detect novel SNPs in the partially tetraploid Atlantic salmon. This strategy may therefore be useful for targeted SNP discovery in several species, and particularly useful in species that, like salmonids, have duplicated genomes.

## Background

Single nucleotide polymorphisms (SNPs) represent the most widespread type of DNA variation in vertebrates. Compared to the commonly used multi-allelic microsatellite markers, the bi-allelic SNPs have much lower polymorphic information content (PIC), but this shortcoming may be compensated using a larger number of SNP markers. Recent technological developments now allow simultaneously detection of several thousands of SNPs at low costs and by use of methods that can be automated and standardized across laboratories [1,2]. As a result, SNPs have become the markers of choice for a range of applications such as QTL detection, gene mapping and parentage assignment. They may also be used as tools for traceability and as genetic markers for conservation management of wild populations [3]. This has led to an increased interest in discovery of novel SNPs in non-model organisms and farmed animals like the economically important Atlantic salmon (*Salmo salar*). If aiming at disclosing economically important traits by fine QTL mapping or to map genes in wild or farmed animals, one would need a large number of validated SNP markers [3,4]. Despite their apparent usefulness as genetic markers, only a moderate number of *Salmo salar *SNP markers have been identified and validated by genotyping in population materials [5-11].

Scanning for new SNPs can be conducted by use of two different approaches [2,4]. When applying the global, or genome wide, SNP discovery approach one aim to detect SNPs randomly in the genome or transcriptome of a given species. The other approach would be a targeted search for SNPs. Using this approach one aims to detect novel SNPs present in a certain set of genes and/or in a certain population of interest (e.g. individuals used in gene mapping or QTL projects) [2]. What approach to use in a given project may be determined by the initial questions that are addressed and, as Lepoittevin et al. pointed out in a recent study, whether a project has a global or targeted approach is important to consider when choosing what SNP discovery method to use [12]. Since the genome wide (or transcriptome wide) approach and the targeted approach are basically different the efficiency achieved when applying one approach cannot be directly compared to the efficiency achieved whith the other approach. Such a comparison would imply measuring how well a method discover a random SNP in the genome to how well a method discover a SNP in a particular gene of interest in a particular population of interest.

Several methods have been suggested for SNP discovery in salmonids [5,6,8-10]. There are, like in many non-model species, no genomic reference sequences in salmonids. The lack of reference genomic sequence data including sequence data from introns limits the number of methods available for detection of novel SNPs in non-model species, particularly if aiming at discovering SNPs in specific genes. It has also been pointed out that SNP discovery in salmonids are particularly challenging since they are partially tetraploid due to a recent genome duplication [5,6,9]. Among the strategies reported as suitable for global detection of novel SNP loci in Atlantic salmon are the *in silico *SNP mining method, and most known SNP markers in *Salmo salar *have been discovered by use of this method [7,9,11]. When applying the EPIC (Exon Primed Intron Crossing) or IPEC (Intron Primed Exon Crossing) methods, on the other hand, one searches for novel SNPs in selected target genes, and these methods thus use a targeted approach [6,13]. The advantage of the latter method is that when priming to less conserved regions (introns) the problem of non-specific amplification of target genes can be reduced, and thereby allow SNP discovery to be carried out in a large proportion of the target genes. The method may therefore, as pointed out by Ryynänen and Primmer [6], be particularly useful for SNP discovery in species that, like salmonids, have large amounts of paralogous sequences. However, the IPEC method depends on reliable sequence data from introns of a given gene to design the locus specific primers needed for SNP detection in that gene. This limits the use of the method for large scale targeted SNP discovery in Atlantic salmon since, like in many other non-model species, there are few genes that have been characterized by genomic sequencing. As a consequence, only a small number of SNPs has been identified by use of the IPEC approach in Atlantic salmon [6].

The *in silico *SNP mining approach [9], although a method used for discovery of the majority of known SNPs markers in Atlantic salmon, also has some minor drawbacks. The *in silico *SNP mining is the initial step of this method and the purpose of this SNP mining is to select a set of putative SNPs that can be validated by genotyping. The SNPs that may be discovered are thus the ones that were present in the individuals used for generating the cDNA libraries. The number of individuals may be relatively low, and the variation present in these individuals (the "*in silico *population") may not be representative of the variation present in other populations of interest. This may not affect the SNP discovery efficiency much if the intention is to discover SNPs randomly in the genome (global approach), but may be important if aiming at detecting novel SNPs in a certain set of genes and/or a certain population (targeted approach). In support of this, results from an evaluation of human SNPs detected by use of a global *in silico *SNP mining approach showed that a large proportion of such SNPs were monomorphic when tested in selected population panels [14].

The economic interest in salmon has led to development of various genomic resources including tissue and developmental stage specific cDNA libraries [15-19]. Partial sequencing of cDNA clones has resulted in a large number of expressed sequence tags (ESTs) from *Salmo salar *made publicly available [20]. These are the ESTs that have been exploited when searching for novel SNPs by use of the *in silico *SNP mining method. Most recent genomic resources made available are the high quality transcript sequence data from full length sequenced cDNAs (FLIcs) [21]. The sequence of each FLIc consists of the coding sequence (CDS) as well as the complete sequence of the 3' untranslated region (3'UTR) of the gene. While there are some short functional sequence motifs located in the 3'UTRs that are conserved, the major part of the 3'UTRs sequences has revealed a much lower conservation level than coding sequences. A low conservation level indicates that the 3'UTR sequences are much less affected than the CDS by the selective forces acting against introduction of variation such as single nucleotide polymorphisms (SNPs) and small deletion-insertion polymorphisms (DIPs) [22]. Thus, not only would the density of SNPs and DIPs be expected to be higher in the 3'UTRs than in the CDSs, but paralog genes would also be expected to be much less similar in their 3'UTRs than in their CDSs. If priming from 3'UTRs in the initial PCR amplification one could, like in the IPEC method, take advantage of the sequence differences in less conserved regions of target genes to limit the problem of non-specific amplification due to large amounts of paralogous sequences. This would be particularly important in the partially tetraploid salmonids. While SNP discovery projects that have applied the *in silico *SNP mining method has exploited ESTs that consist of a combination of CDS and UTR sequences, a UTR-primed method that utilizes sequence information from annotated FLIcs could target 3'UTRs only, and thus, allow for a SNP search in gene fragments that are expected to have a higher SNP density than the ESTs utilized so far. Theoretically, both 5' and 3'UTRs could be searched for SNPs. However, the 5'UTRs in salmon cDNA inserts are considerably shorter than 3'UTRs and thus, less useful for SNP mining [21]. Taken together, this indicates that a method that utilizes FLIcs to target 3'UTRs may be a suitable SNP discovery method for targeted SNP detection in salmonid genes. High quality sequenced FLICs represent the most useful transcript sequences, and this has led to large scale sequencing of full length insert cDNAs in several non-model species (e.g.[21,23-26]). A strategy that successfully utilize the 3' UTR sequence information from FLIcs for targeted SNP discovery would therefore be of general interest.

In this study we have evaluated the performance of two SNP discovery methods applied for targeted SNP discovery. We present results from testing the UTR-primed method for SNP discovery when targeting 3'UTRs in a set of full length cDNA (FLIc) genes. Due to the successful use of the *in silico *SNP mining method for global SNP discovery we have included this method in our study, and we present results from testing the *in silico *SNP mining method when applied for targeted SNP discovery in FLIc genes. We also present more than 100 novel SNPs discovered in well characterized and annotated *Salmo salar *genes (type I markers), including some SNPs that may be of functional importance.

## Results and Discussion

### Evaluation of methods for targeted SNP discovery

The target genes used for testing SNP discovery efficiency in this study were selected from a white muscle tissue and pre-smolt developmental stage specific cDNA library [19]. The genes have been characterized by full length sequencing of cDNAs followed by annotation of coding sequences (CDS) and untranslated regions (UTRs). In most cases the full length sequenced cDNAs (FLIcs) consist of the complete coding sequence (cCDS), and in all cases they consist of the complete 3'UTRs of the target gene [21]. Excluding ribosomal protein genes, a total of 246 target genes were consecutively selected from [21] and used to test the methods. Sixty genes were tested by use of both methods while additional 146 (total of 208) and 98 (total of 158) genes were used for testing the 3'UTR-primed and *in silico *SNP mining discovery methods, respectively. The small population material that was targeted for SNP discovery consisted of the four parental individuals in our reference mapping families, two individuals selected from aquaculture stocks and four individuals from Norwegian rivers (wild salmon). All individuals were genotyped in all candidate SNPs tested.

**Figure 1:**
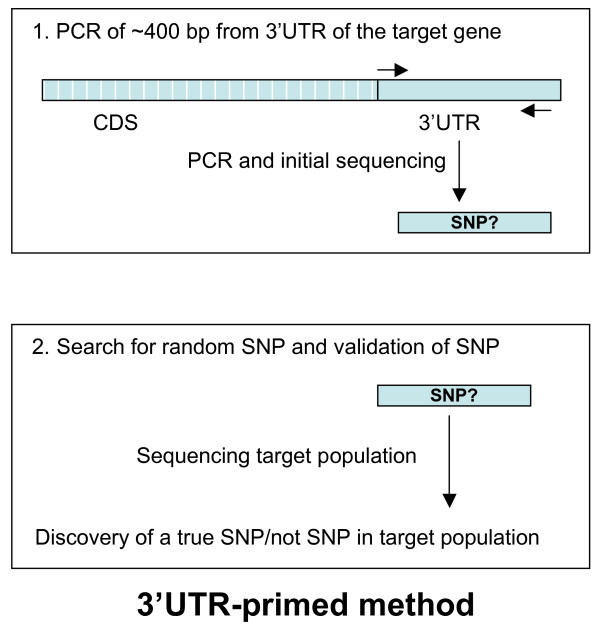
**The figure shows the two main steps of the UTR-primed method**. Step 1 is the PCR and initial sequencing step where a fragment of approximately 400 bp from the 3'UTR of the target gene was amplified and sequenced. Step 2 illustrates the sequencing of the PCR fragment from step 1 in all individuals in the target population. This step served two purposes: a search and identification of a SNP and at same time a validation of the identified SNP as a true SNP (not a PSV).

The UTR-primed SNP discovery method consisted of two main steps (Figure 1), and the final success rate of the method depended on the success rate of each of these consecutive steps. The first step was a PCR amplification of the 3'UTR-derived fragment from the target gene followed by sequencing the amplified fragment to assure that the PCR fragment had the necessary quality needed for the targeted SNP discovery (step 1, UTR-primed method, Figure 1). The second step was the genotyping (sequencing) of the UTR-primed PCR product in a small population material (step 2, UTR-primed method, Figure 1). The latter step served as both a SNP discovery step as well as a validation step that could exclude paralogous sequence variants (PSVs).

In the first step a pair of PCR primers targeting sequences within the 3'UTR (UTR-primed PCR) was designed for each of the target genes to amplify a fragment of about 400 bp (see methods). Amplification of such fragments was performed by use of genomic DNA from two individuals followed by agarose gel analyses of the PCR products. If the PCR amplification resulted in a single clear band in the agarose gel test, the PCR products were further analysed by DNA sequencing. All target genes where the initial PCR amplification resulted in either no PCR products (negative or weak bands on agarose gel testing), multiple PCR products (multiple bands on agarose gel) or revealed multiple heterozygote sites (five or more) when analysed by DNA sequencing were not included in the next main step. Twenty-three of the 208 selected primer pairs failed on the agarose gel test while an additional twenty of the selected primer pairs failed on the sequencing test. Thus, in this initial PCR and sequencing step 43 UTR fragments from selected target genes where not amplified properly or lacked the desired sequence quality while in 165 genes (79%) the primer pairs produced PCR fragments that could be further used for SNP discovery.

All the 165 target genes that passed the first step were included in the second step aiming at detecting novel SNPs in the UTR-primed PCR products. In this step individuals from the small population material were genotyped by sequencing the amplified target gene fragments. PCR products were generated by amplification of the individuals genomic DNA and the chromatograms from sequencing of individual samples were inspected manually to assure that heterozygote positions were detected. Finally, all chromatograms in the population panel were aligned and compared. A given site within the sequence was accepted as a true polymorphism only if the rare variant was observed in at least two individuals. These comparisons resulted in the discovery of at least one SNP or DIP in 73 of the 165 genes. This gives a SNP discovery rate of 44% in this second step of the UTR-primed method. A summary of the success rate in each of the main steps is given in table 1. Taken together, one (or more) bi-allelic polymorphism was discovered in 36% of the 208 genes initially selected for testing of the UTR-primed SNP discovery method (table 1, SNP discovery success rate). Sixty target genes were tested by both methods, and a SNP was discovered in 14 of these 60 genes (23%) when applying the UTR-primed method

**Table 1:** Summary of success rates when testing two strategies for SNP discovery in a set of target genes

SNP discovery strategies tested:	3'UTR-primed SNP discovery approach	*in silico *SNP mining approach
Number of genes (cCDS-FLIcs) tested	208	168
candidate SNPs success rate^1^	-	0.82 (131/168)
Initial PCR and sequencing success rate^2^	0.79 (165/208)	0.47 (61/131)
SNP discovery rate^3^	0.45 (74/165)	0.41 (25/61)
SNP discovery success rate^4^	0.36 (74/208)	0.15 (25/168)

^1^Number of target genes with at least one putative SNP/total number of target genes selected for testing the in silico SNP mining method. ^2^Number of primer pairs producing PCR fragments suitable for sequence analysis/total number of primer pairs tested. ^3^SNP discovery rate: one or more SNP discovered/PCR fragments analysed in the small population materials. ^4^SNP discovery success rate: genes with at least one SNP discovered/total number of genes tested.

The comparisons of individual genotypes also served as a validation step where paralogous sequence variants (PSVs) could be excluded. A PSV located in highly similar paralogous sequences can not be distinguished from a true SNP based on results from one or a few individuals. However, while a true SNP segregates according to Hardy/Weinberg expectations in a population panel, a PSV would appear as a heterozygote in all individuals [27]. Any "SNP" revealing such heterozygous excess (a significant deviation from H/W expectations) was not included in the final set of validated SNP markers given in table 1.

**Figure 2:**
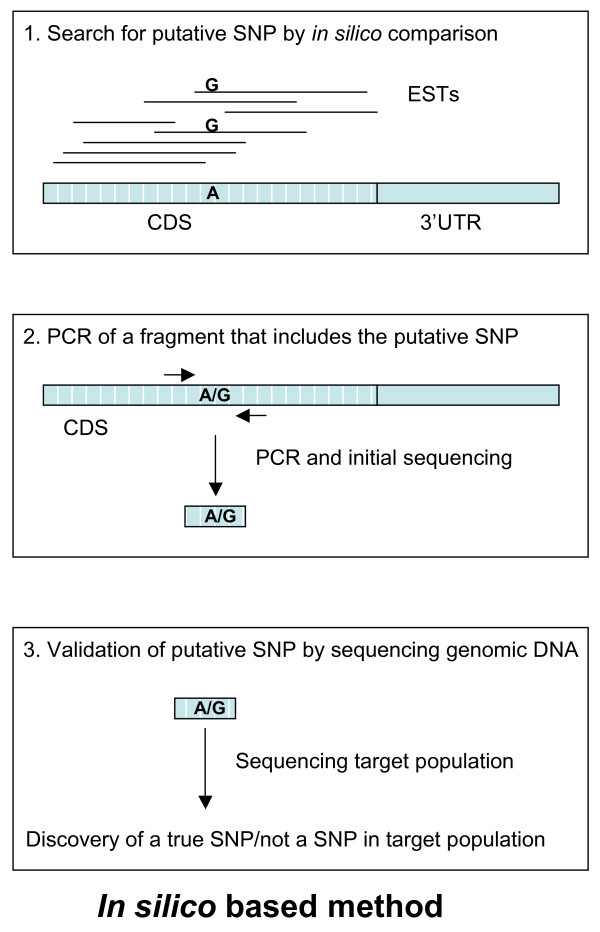
**The figure shows the three main steps of the in silico method**. Step 1 illustrates alignment of ESTs to the FLIcs from the target gene. This is the step where putative SNPs could be identified. Step 2 shows the PCR and initial sequencing step where a fragment that included the putative SNP was amplified and sequenced. Step 3 illustrates the sequencing of the PCR fragment from step 2 in all individuals in the target population to validate the putative SNP as a true SNP.

We performed a parallel testing of the *in silico *SNP-mining method in 158 target genes, sixty of these also tested by use of the UTR-primed SNP discovery method. The *in silico *SNP mining method included the two steps of the UTR-primed method (step 2 and 3, *in silico *method, Figure 2), but also had a prior step that aimed to identify candidate SNPs by comparisons of transcript sequence data *in silico *(step 1, *in silico *method, Figure 2).

The *in silico *comparisons (candidate SNP mining) were performed manually by comparing the FLIcs to the ESTs available from GenBank that were likely to represent target gene transcript sequences (see methods). The chromatograms from such ESTs were aligned to the corresponding FLIcs and the alignments were manually searched for putative SNPs. Any sites that revealed sequence differences at the 50 first or 50 last nucleotides in a given EST when compared to the corresponding FLIc were not considered as putative SNPs. Also, if a given EST revealed multiple sequence differences within the sequence (e.g. >3 differences for each 100 bp) when compared to the corresponding FLIc the EST was not considered as originating from an allelic sequence, but rather to represent paralogous sequence. Consequently, any sequence differences in such ESTs were not included as putative SNPs in this initial step. The *in silico *SNP mining step resulted in discovery of 131 putative SNPs in the 158 target genes (table 1, candidate SNP success rate 0.82).

Primers were then designed to amplify a short sequence (in average 150 bp) that included the candidate SNP. The PCR products from these amplifications were tested by agarose gel analysis and subsequently sequenced. The results from the initial PCR and sequencing step showed that 70 of the 131 PCR products from target genes failed in either the PCR amplification or sequencing quality test of PCR products. Thus, only 61 PCR products (47%) from 57 different target genes could be further used for SNP search in the population material.

The final SNP discovery step was, like in the UTR-primed method, performed by genotyping individuals in a small population material. All 61 PCR products passing the previous quality step was tested in this final step, and a SNP was identified in 25 out of the 61 PCR products. This gives a SNP discovery rate of 41% (table 1). Taken together, we were able to detect 25 novel SNPs from the set of 158 target genes initially selected. This gives a SNP discovery success rate of 15% (table 1) when using an *in silico *based approach for targeted SNP discovery. A SNP was discovered in 6 cases (10%) when applying the *in silico *SNP mining method for SNP discovery on the 60 target genes tested by both methods.

In this study we wanted to test methods suitable for a targeted SNP discovery approach in Atlantic salmon. Initially, we therefore also wanted to test both the EPIC (exon-primed intron crossing) and the IPEC (intron-primed exon crossing) strategies [6,13]. We tested a few selected genes by using the EPIC approach. However, this approach showed a very poor initial PCR and sequencing success rate (data not shown). These results were in agreement with findings in Ryynänen and Primmer [6] showing similar poor initial amplification success rates (about 10% when amplifying fragments of 400 bps). This indicates that using larger amplicon sizes when priming from coding sequences, e.g. generating amplicons larger than the 150 bp fragments used in the *in silico *method, would lower the success rate of the initial PCR. Although we did identify a few SNPs using the EPIC method, no further testing was carried out due to the poor success rate in the initial step of this method.

All the target genes were selected from FLIcs and have been characterized by full length sequencing of the corresponding cDNAs. However, the complete genomic sequence of the target genes had not been characterized, and the intron sequence data needed to design primers for testing the IPEC (intron-primed-exon-crossing) method [6] was therefore not available. Thus, a direct comparison with the IPEC method was not possible to carry out. The advantage of the IPEC method for SNP discovery, compared to other methods, has been the high success rate achieved in the initial amplification step. Even if a direct comparison between the IPEC and UTR-primed methods could not be carried out it was possible to compare the success rate of the initial PCR and sequencing step of the UTR-primed method to the reported success rate of the IPEC method. In Ryynänen and Primmer [6] the initial PCR and sequencing success rate was reported to be 0.77 when applying the IPEC method. In the present study the UTR-primed SNP discovery method showed a success rate of 0.79 (Initial PCR and success rate, table 1). This showed that the two methods had similar success rates in the initial amplification step. The *in silico *SNP mining method had a much lower success rate at this step (0.47, table 1). There may be several explanations to this difference in success rate between the two methods. One reason could be that many putative SNPs were located in coding sequences. In the group of target genes tested by the *in silico *method 88% of all putative SNPs were located in coding sequences and 95% of putative SNPs that failed in the initial amplification step when applying this method were located in coding sequences. When amplifying fragments from coding sequences the primers would be directed at the more conserved region of the target genes, and, as a consequence of this, have a higher risk of amplifying non-specific PCR products (e.g. paralogous sequences). Other reasons that the initial PCR amplification failed more often when testing the *in silico *SNP mining method could be that primers made using transcript sequences by chance were located in exon-intron boundaries, or that large introns were included in the amplicon leading to a poor amplification efficiency.

In a study by Smith et al. [10] a method for discovering SNPs in various Pacific salmon species was tested. Sequence data from rainbow trout and Atlantic salmon was used to target particular loci in the Pacific salmon species of interest and a targeted SNP discovery approach was used. They were able to successfully genotype and validate SNPs in about 18% of the target loci when investigating a target population consisting of 50 individuals. The UTR-primed method used in our study seems to be a better option for targeted SNP discovery with a success rate of 36% when tested in 10 individuals only.

A conversion rate is often used for measuring the performance of the *in silico *SNP mining of putative SNPs (first step of the *in silico *method). The conversion rate refers to the number of polymorphic SNPs confirmed by genotyping divided by the total number of putative SNPs discovered in the SNP mining [12]. Several studies have showed that this conversion rate may differ substantially (from about 30-70%) depending on the quality of the EST data used for *in silico *SNP mining as well as on the species that are investigated [12,28,29]. If there is a large amount of sequence errors in the EST data this may appear as false positive putative SNPs. Highly similar paralogous sequences could also lead to inclusion of false positive putative SNPs (that in fact are single basepair differences between paralogs, PSVs). Finally, if the *in silico *data is not representative of the population used in the genotyping, the putative SNPs selected may be monomorphic in the population used for validation. Vice versa, SNPs present in the genotyped population, but not in the *in silico *data will not be discovered since these SNPs don't appear as putative SNPs in the first place. All the mentioned factors may lead to substantial decrease in the conversion rates. When applying the *in silico *SNP mining method for global SNP discovery in Atlantic salmon a dedicated pipeline for selection of putative SNPs have been introduced to maximize the conversion rate [11]. Genotype validations of the highest ranking putative SNPs selected by use of this pipeline showed a conversion rate of about 70%. In our study, when disregarding the fragments that could not be genotyped, we achieved a conversion rate of 41% (25 SNPs in the 61 fragments with putative SNPs that could be genotyped, table 1). One explanation to this large difference between conversion rates could be that the *in silico *SNP mining was performed manually in our study while a dedicated pipeline was used to select the putative SNPs in Hayes et al., and only the SNPs with best PolyBayes ranking were genotyped [11]. It is likely that the dedicated pipeline more efficiently eliminated false positive SNPs than the manual selection used in our study. However, we applied a targeted approach, and if we had used a more strict selection of putative SNPs this would, in our case, decreased the total number of putative SNPs rather than led to selection of other putative SNPs in the target genes that were more likely to pass the genotype validation. Thus, while applying such a dedicated pipeline could have been less time consuming, we do not believe that this would have led to a large increase in the number of SNPs discovered in the target genes. Another difference was that a total of 65 individuals from different populations were tested in the genotype validation step in Hayes et al., while a smaller number of individuals were included in this study (only 10 individuals tested for each putative SNP). Thus, while low frequency SNPs or population specific SNPs present in the *in silico *data may have been detected in Hayes et al, such SNPs may have appeared as monomorphic in our validation. Taken together, the low conversion rate leading to fewer putative SNPs that were validated as true SNPs in our study than in Hayes et al. could be a consequence of using a targeted SNP discovery approach (targeting particular genes and using a small target population).

In conclusion, there was a significant difference in total SNP discovery efficiency between the two methods tested in our study. The UTR-primed method was the one that performed better for a targeted SNP discovery approach in our hands. While a novel SNP was detected in 36% of target genes applying the UTR-primed method the *in silico *SNP mining method detected a novel SNP in 15% of target genes (table 1). In the set of target genes tested by both methods (n = 60) a SNP was discovered in 14 cases (23%) by use of the UTR-primed method and in 6 cases (10%) when applying the *in silico *SNP mining method. The UTR-primed method was also the less labour intensive and time consuming one since the initial SNP mining step was omitted.

### Characterisation of SNPs discovered in salmon genes

A total of 112 SNPs or DIPs were discovered in 88 different genes in this study. A single SNP (or DIP) was discovered in 70 of the genes while two and three SNPs were discovered in 12 and 6 genes, respectively. All SNPs have been submitted to dbSNP http://www.ncbi.nlm.nih.gov/projects/SNP/ in GenBank [dbSNP: ss179319972-179320081, ss250608647-250608648]. A complete overview of all SNPs (and DIPs) discovered, the dbSNP NCBI assay ID (ss) accession numbers as well as the corresponding FLIc accession numbers from GenBank is given in additional file 1. These are the first SNPs discovered in well characterized *Salmo salar *FLIcs and thus, are an important contribution to the number of validated SNP markers in Atlantic salmon. All polymorphisms discovered by the UTR-primed method were located within the 3'UTRs of the target genes. The SNPs discovered by use of the *in silico *SNP mining method were located both in exons, introns and in 3'UTRs while the few SNPs discovered by use of the EPIC method were located in introns. table 2 gives a summary of the target genes where SNPs were discovered in the coding sequence. Mapping the exact location of the substitutions within the reading frame showed that five of the changes are synonymous substitutions while three of the substitutions represent missense substitutions resulting in allelic variants coding for different amino acids.

**Table 2:** SNPs in coding sequences

Gene:	dbSNP ss #	Genbank acc #	Type of substitution
ribosomal protein s27	179319983	BT043888.1	synonymous
ribosomal protein s27	179319984	BT043888.1	missense
malate dehydrogenase	179320008	BT043833.1	synonymous
Glutamyl tRNA aminotransferase-like	179319990	BT043526	missense
KRAB box and zinc finger C2H2 type domain	179319994	BT043939.1	synonymous
KRAB box and zinc finger C2H2 type domain	179319995	BT043939.1	synonymous
KRAB box and zinc finger C2H2 type domain	179319996	BT043939.1	synonymous
Proteasome 26s subunit non-ATPase 9	179320005	BT043553	missense

Applying the UTR-primed method we have scanned a total of 66000 bp by sequence analysis of 165 UTR derived PCR products and discovered a total of 63 SNPs and 16 DIPs. This indicates that there is about one bi-allelic polymorphism in every 840 bp of the 3'UTRs. The population material screened when searching for SNPs in the 165 PCR fragments consisted of relatively few individuals. Thus, it is likely that most rare SNPs or population specific SNPs in the target genes were not discovered in this study. In Ryynänen and Primmer a population material consisting of a similar small number of individuals were screened when searching for SNPs [6]. Comparing the SNP densities reported for introns and coding sequences in Ryynänen and Primmer shows that the SNP density observed in the 3'UTRs in our study (1 in 840 bp) are, as expected, higher than the SNP density in coding sequences (1 in 1450 bp) but lower than the SNP density in introns (1 in 400 bp).

A total of 62 SNPs that were shown to be polymorphic in one or more of the SALMAP parents where genotyped in the reference families [30]. The 62 genes that contained the SNPs were mapped on to our existing Atlantic salmon map (Hoyheim et al in prep). The new SNPs covered 26 of the 29 linkage groups on the map, no new markers were added to linkage groups 11, 19 and 27. These markers increased the number of genes on our current map from 25 to 87.

## Conclusion

FLIcs are important genomic resources that have been developed in many farmed animals. The UTR-primed SNP discovery strategy successfully utilized data from full length insert cDNAs (FLIcs) for targeted SNP discovery in *Salmo salar *genes. All cDNA clones used for developing EST resources have inserts consisting of complete 3'UTRs. The sequence of the 3'UTR in any target gene identified by its EST sequence may therefore be relatively easily accessed by sequencing the cDNA insert from the 3' end. The UTR-primed SNP discovery approach may therefore be the better strategy for targeted SNP discovery in non-model species, and may be particularly useful in species, that like salmonids, has duplicated genomes. Detection of SNPs by use of a global approach and the *in silico *SNP mining method has proven to be a successful strategy for discovery of novel SNPs in many species. However, if using small populations or fine mapping certain regions or genes, a targeted approach may more efficiently provide the novel SNPs necessary for that particular project. Thus, we believe that the targeted UTR-primed method may be a useful complementary method for SNP detection in farmed animals and non-model species.

## Methods

### Population material

A material consisting of the four parental individuals in the SALMAP reference families, two individuals from aquaculture stocks and four individuals from Norwegian rivers, a total of 10 individuals, were used as a panel for genotype validation of SNPs. All loci were typed in these 10 individuals. If a SNP was revealed in only one of the ten individuals, an additional typing of this marker was performed in five individuals. A novel candidate SNP was only approved as a true SNP if the rare variant was observed in at least two individuals. All new SNPs discovered in this study were submitted to the dbSNP database http://www.ncbi.nlm.nih.gov/snp. In all cases where a novel SNP was discovered in the parental individuals in the SALMAP reference families genotyping of the whole family was performed (two parents and 46 offspring in each of two families).

### Selection of target genes

The genes selected for testing the SNP discovery methods were those expressed in white muscle tissue at the pre-smolt developmental stage. They were chosen as target genes because they have all been characterized by high quality full length sequencing of their transcript sequences including the complete 3' UTRs [21]. Apart from avoiding some genes that were small (<500 bp), mostly small ribosomal proteins, the putative function of the genes were not considered when selecting target genes. A total of 158 target genes were used to test the *in silico *SNP discovery method while 208 target genes were used for testing the UTR-primed SNP discovery method. A total of 60 genes were tested using both methods. The SNPs identified in this study were used for gene mapping if informative in our family materials. To make an additional independent comparison of the methods and at same time minimize cost and labor when discovering suitable SNPs for mapping target genes, the additional genes included in this study were randomly divided in two groups where each group were tested by one method only.

### PCR amplification and DNA sequencing

All primers were designed by use of primer 3 software http://frodo.wi.mit.edu/primer3/[31]. When testing the *in silico *SNP mining method the primers for the initial PCR were designed with short product size range settings (130-170 bp) in an attempt to limit PCR failure due to co-amplification of large introns. When testing the UTR-primed SNP discovery method the primers for the initial PCR were designed with product size range settings 350-450 bp to amplify fragments that could be completely sequenced in one direction (all fragments were sequenced in both forward and reverse direction). When designing primers to test the EPIC method the product size range settings were 200-250 bp. The primers for the EPIC method were designed with such a size range setting in an attempt to include introns (intron crossing) when using genomic DNA as template. Except the modification of the PCR product size range settings, all other settings, including Tm optimum of 60°C, were the default conditions given by primer3 software. The -21m13 forward (5'TGTAAAACGACGGCCAGT3') and -21m13 reverse (5'CAGGAAACAGCTATGACC2') universal primer sequences were added to the 5' end of forward and reverse PCR primers, respectively. The PCR reactions were performed using standard conditions: 50 ng genomic DNA, 1.5 mM MgCl, 0.2 mM dNTP's, 0.5 μM of forward and reverse primer, 5% DMSO and 0.5 U *Taq *polymerase (Quiagen) in total volumes of 25 μl. A Tetrad Thermal Cycler (PTC-225, MJ Research) was used for the PCR amplification with the following programme: 94°C for 3 min, then 28 cycles with initial denaturation at 94°C 30 s, annealing 56°C 30 s, extension 72°C 2 min and a final extension step at 72°C for 5 min.

DNA sequencing was performed by use of forward and reverse -21m13 universal primers and the MegaBACE 1000 automatic sequencer (Amersham Pharmacia) as described in Adzhubei et al. [19]. The individual chromatograms were manually inspected and evaluated to ensure that only high quality sequences were used in the SNP discovery analyses. The individual sequences were aligned and compared by use of Sequencher 4.7 software. Candidate SNP loci were approved as true SNPs only if (I) the sequence spanning the SNP was of high quality and (II) the rare variant was observed in at least two individuals. Paralogous sequence variants were eliminated by testing for heterozygote excess. If a SNP revealed a "heterozygote" genotype in all the ten individuals tested this represents a significant deviation from Hardy-Weinberg expectations (P value = 0.001). "SNPs" that revealed such distributions (heterozygote excess) was regarded as paralogous sequence variants (PSVs) resulting from co-amplification of paralogous sequence and not included in our validated SNPs.

### Identification of candidate SNPs by *in silico *SNP mining

The inital step of the *in silico *SNP mining method was the selection of Atlantic salmon transcript sequences (ESTs) from GenBank that were likely to originate from the selected target genes. An initial BLASTN homology search against salmonid EST data in GenBank using each of the selected FLIcs as input was performed to identify such candidate sequences. The Atlantic salmon EST transcript sequences that was finally selected for further comparison to the FLIcs were those matches that returned E-values less than 10^-20 ^and with at least 96% sequence identity to the target genes. Only ESTs where chromatograms were available was used in the *in silico *comparison ([19] and http://web.uvic.ca/grasp/). The selected ESTs were compared to the corresponding FLIcs using Sequencher 4.7 software. These alignments were manually inspected and obvious sequence errors caused by low quality data, sequence differences located within poly G or C sequences or other simple repeats were not considered as putative SNPs. The quality of EST data is generally low at the very beginning and the very end of the chromatograms. In order to process all ESTs in the same manner and to avoid too many sequence errors included as putative SNPs any difference within the 50 first and 50 last base pairs in the EST sequences were not considered as putative SNPs. Similar settings were used in Hayes et al. [9]. Only sequence differences within the ESTs that unambiguously could be classified as to be sequences of good quality in the visual inspections were accepted as candidate SNPs. Some EST sequences revealed multiple sequence differences e.g. more than 4 differences within a 100 bp region of the alignments. These differences were not considered as multiple candidate SNP loci even if the sequence met our quality standard. Instead, such ESTs were considered as originating from highly similar paralogs of the target gene. When a candidate SNP was detected in the manual *in silico *SNP mining primers were designed to amplify a fragment of the target gene that included this candidate SNP as described in the PCR amplification and DNA sequencing section of methods.

### Testing for significant differences between SNP discovery methods

To test whether there was a significant difference in SNP discovery efficiency between the two methods the number of SNPs detected in the two sets of target genes was examined by use of a 2 × 2 contingency table. A chi square test was used to test the null hypothesis that there was no significant difference in number of SNPs detected in the sets of target genes using each of the two methods. These tests revealed significant differences between the two methods when comparing all SNPs detected in all target genes (p = 0.001), or if only comparing SNPs detected in the 60 target genes tested by use of both methods (p = 0.028).

## Authors' contributions

RA participated in PCR amplification and sequencing when testing the methods, performed the search for putative *in silico *SNPs, loaded all SNP data to GenBank, carried out bioinformatics and statistics, and drafted the manuscript.

BH conceived and coordinated the study, performed the linkage analysis and helped to draft the final manuscript.

SL performed major part of the PCR and sequencing and helped in the manual processing of the data.

All authors have read and approved the final manuscript.

## Supplementary Material

Additional file 1 **A complete overview of all SNPs and DIPs discovered, the dbSNP submitter (ss) accession numbers as well as the corresponding FLIc accession numbers from GenBank**. Click here for file
